# Dr. Laird M. Woods

**Published:** 1906-03

**Authors:** 


### OBITUARY NOTES.

Dr. Laird M. Woods, of Dallas, Oregon, died at his home February 12, 1906, of septicemia, resultant from a carbuncle, aged 70 years. He formerly served as coroner of Polk County, in which Dallas is situated, and before that as burgess of Wheatland, Pa., for several terms, when he resided in that place. He was also for ten years Surgeon of the Pennsylvania railroad. He graduated from the University of Buffalo in 1872.

